# Morphological examination of pelvic floor muscles in a rat model of vaginal delivery

**DOI:** 10.1186/s12884-024-06278-5

**Published:** 2024-01-31

**Authors:** Yui Abe-Takahashi, Takeya Kitta, Mifuka Ouchi, Hiroki Chiba, Madoka Higuchi, Mio Togo, Naohisa Kusakabe, Hidehiro Kakizaki, Nobuo Shinohara

**Affiliations:** 1 Department of Renal and Genitourinary Surgery, Graduate School of Medicine, Hokkaido University, Sapporo, Japan; 2 Department of Physical Therapy, Faculty of Health Sciences, Hokkaido University of Science, Sapporo, Japan; 3 Department of Renal and Urologic Surgery, Asahikawa Medical University, Asahikawa, Japan

**Keywords:** Morphological change, Muscle fiber composition, Pelvic floor muscles, Stress urinary incontinence, Urethral function, Vaginal distention

## Abstract

**Objective:**

This study investigated morphological changes in the composition of the pelvic floor muscles, degree of atrophy, and urethral function in a rat of simulated birth trauma induced by vaginal distension (VD) model.

**Methods:**

Female Sprague–Dawley rats were classified into four groups: a sham group, and 1, 2, and 4 weeks post-VD (1 W, 2 W, and 4 W, respectively) groups. We measured the amplitude of urethral response to electrical stimulation (A-URE) to evaluate urethral function. After measuring the muscle wet weight of the pubococcygeus (Pcm) and iliococcygeus (Icm) muscles, histochemical staining was used to classify muscle fibers into Types I, IIa, and IIb, and the occupancy and cross-sectional area of each muscle fiber were determined.

**Results:**

There were 24 Sprague–Dawley rats used. A-URE was significantly lower in the 1 W group versus the other groups. Muscle wet weight was significantly lower in the VD groups versus the sham group for Pcm. The cross-sectional area of Type I Pcm and Icm was significantly lower in the VD groups versus the sham group. Type I muscle fiber composition in Pcm was significantly lower in the VD groups versus the sham groupand lowest in the 2 W group. Type I muscle fiber composition in Icm was significantly lower in the 2 and 4 W groups versus the sham group.

**Conclusion:**

Muscle atrophy and changes in muscle composition in the pelvic floor muscles were observed even after improvements in urethral function. These results may provide insight into the pathogenesis of stress urinary incontinence after VD.

## Background

Pelvic and perineal skeletal muscles are involved in both reproductive and lower urinary tract functions. Moreover, compared with males, females are more susceptible to pelvic floor dysfunctions because of their close association with factors (e.g., aging, menopause, and number of vaginal deliveries). A large number of vaginal deliveries leads to the weakening of the pelvic floor muscles and the onset of stress urinary incontinence. It has been reported that the prevalence of stress urinary incontinence in individuals who have had a vaginal delivery is 12.2%. This rate is 2.4-fold higher than that recorded in individuals who have undergone cesarean Sect. [1]. Furthermore, stress urinary incontinence after vaginal delivery persists for > 2 months in approximately 2% of cases [2]. 

Simulated birth trauma has been shown to induce stress urinary incontinence and urethral dysfunction in rats with vaginal distention (VD) [3]. In a single VD model, stress urinary incontinence occurred in all rat model of VD at 4 days postoperatively, with significant decreases in urethral baseline pressure (UBP) and amplitude of the urethral response to electrical stimulation (A-URE) [3]. 

In humans, the pubococcygeus muscle (Pcm) reaches a maximal stretch ratio of approximately 3 during simulated vaginal birth, and nerve damage to the anorectal levator ani muscle is observed [4, 5]. Therefore, VD negatively affects the structure and function of these muscles.

Changes in morphology and the distribution of fiber types after nerve transection have been reported in skeletal muscles [6]. The Pcm consists of heterogeneous proportions of slow-twitch oxidative fibers (Type I), fast-twitch oxidative glycolytic fibers (Type IIa/d), and fast-twitch glycolytic fibers (Type IIb) [7]. Pelvic floor muscles are mainly composed of two types of fibers, playing distinct roles in pelvic physiology. Type I fibers mainly support the pelvic organs because they can maintain tension for an extended period of time, whereas Type IIa fibers contract quickly [8]. 

In a previous study, decreased urethral resistance was observed in rats that had undergone bilateral transection of the somatomotor branch of the pelvic nerve to the Pcm [9]. Thus, the pelvic floor muscles play important roles in the function of the lower urinary tract.

Currently, the effect of VD on pelvic floor muscles composition remains unknown. Peripheral nerve injury causes denervation and a wide variety of muscle disorders, such as atrophy [10]. Therefore, we hypothesized that VD would cause atrophy in the pelvic floor muscles and alter the distribution of muscle fiber types. The objective of this investigation was to determine morphological changes in the composition of the pelvic floor muscles, degree of atrophy in the pelvic floor muscles, and urethral function in a rat model of VD.

## Methods

### Animals

Female Sprague–Dawley rats (initial body weight : 211.0–328.3 g; age: 12 weeks) were used. The rats had *ad libitum* access rat chow and water, and were treated according to the National Institutes of Health (Bethesda, MD, USA) guidelines. The experiments conducted in this study were approved by the Animal Experimentation Committee of Hokkaido university (No. 019–0062). The rats were classified into groups as follows: (1) sham-operated group (sham group); (2) 1-week post-VD group (1 W group); (3) 2-week post-VD group (2 W group); and (4) 4-week post-VD group (4 W group). The sample size calculations were based on reported sample size calculations in animal studies for a one-way ANOVA design [11]. The following two formulas; Minimum *N* = 10 / *k* + 1, and Maximum *N* = 20 / *k* + 1 (*N* = total number of subjects, *k* = number of groups, and *n* = number of subjects per group) allow the sample size to be determined. In this study, the formula based on Maximum *N* (Maximum *N* = 20 / *k* + 1) was used.

### VD

After cutting off the tip, a 14-Fr Foley balloon catheter (Clinique, Yokohama, Japan) was inserted into the vagina under anesthesia, and the vaginal opening was closed with sutures to prevent catheter dislodgement. Subsequently, the balloon was distended with 4 mL of water for 4 h to cause obstruction of the urethral [12]. In the sham group, the catheter was inserted in the vagina; however, the balloon was not inflated.

### Measurement of urethral pressure

Urethral pressure measurements were performed 2 weeks after sham surgery in the sham group, as well as at 1, 2, and 4 weeks after VD in the 1 W, 2 W, and 4 W groups, respectively. To eliminate reflex bladder contractions, both ureters were ligated; this was followed by bilateral transection of the vesical branches of the pelvic nerves near the internal iliac vessels. A 3.5-Fr nylon catheter (SPR-524; Millar Instruments, Houston, TX, USA) tipped with a microtransducer 1 mm from the tip and connected to a pressure control unit (PCU-2000; Millar Instruments) was inserted into the middle urethra at 12.5–15 mm from the urethral orifice. The urethral response was monitored using an analog-to-digital converter (Power Lab®; AD Instruments, Nagoya, Japan). Data were recorded on a personal computer at a sampling rate of 400 Hz. An isolator (SS-104 J; Nihon Kohden, Tokyo, Japan) and an electrical stimulator (SEN-3301; Nihon Kohden) were used to stimulate the exposed bilateral oblique abdominal muscles (duration: 0.5 ms; intensity: 1.8–2.0 V; 20-ms intervals; repeated every 10 s). For electrical stimulation, bilateral incisions were performed around the 11th to 13th ribs to insert the electrodes. These incisions provided access to the oblique abdominal muscles. The A-URE, which was defined as the maximum change in pressure (cmH_2_O) from baseline, and UBP, which was determined from the flat area immediately before the pressure recording in response to electrical stimulation, [9] were also measured. During the measurements, the rats were placed in the supine position and anesthetized.

### Collection of Pcm and iliococcygeus muscles (Icm)

After the UBP and A-URE measurements, the target Pcm and Icm were harvested by inserting scissors through the pubic symphysis to open the iliac crest under urethane anesthesia. All animals were euthanized by exsanguination under deep anesthesia with 3% isoflurane inhalation. Fat and connective tissue were removed from the Pcm and Icm, and the muscle wet weight was measured using a digital scale. To obtain cross-sections, the Pcm and Icm were vertically mounted on a cork plate in tragacanth gum jelly, frozen by immersion in isopentane solution in liquid nitrogen, and stored at − 80 °C.

### Histologic and histochemical analysis

Frozen Pcm and Icm samples were subjected to histochemical analyses. The frozen cross-sections were cut (thickness: 10 μm) using a cryostat (CM 1510 S; Leica Microsystems, Wetzlar, Germany) cooled to − 20 °C. Two serial sections were thawed and air-dried for 30 min. Cryosections were stained for myosin adenosine triphosphatase (ATPase; pH 10.2) reaction and succinate dehydrogenase (SDH) activity, as previously reported [13]. Consecutive sections were processed for myofibrillar ATPase after alkaline pre-incubation according to a previous study [14]. Next, ATPase activity was utilized to separate the fibers into two major categories, namely Type I and Type II. The fast-twitch fibers were subsequently divided into two groups based on their staining intensity after an SDH activity assay, namely Type IIa and Type IIb. For SDH staining, all sections were incubated for 30 min in a combination of sodium succinate, phosphate buffer, and nitro blue tetrazolium at 37 °C. The SDH- and alkaline ATPase-stained sections were subsequently observed at 100× using a light microscope (BX 43; Olympus, Tokyo, Japan). A digital camera attached to the microscope (FX 630; Olympus) was used to capture photomicrographs of each muscle section. For each muscle, a computer-aided image processing system (ImageJ version 1.52a, National Institutes of Health) based on SDH- and alkaline ATPase-stained images was used.

The fibers were classified into: Type I (slow-twitch oxidative); Type IIa (fast-twitch oxidative glycolytic); and Type IIb (fast-twitch glycolytic). In addition, the cross-sectional area (CSA) of 100 randomly selected fibers of each type was measured. Both Pcm and Icm specimens on the left side were used for the analysis.

### Statistical analysis

The R software package (version 4.1.3) was used. Comparisons for UBP, A-URE, muscle wet weight / body weight (mg/g), (Pcm, Icm), type of myofiber composition, and each CSA among the four groups were analyzed by one-way analysis of variance (ANOVA), Welch’s *t*-test, and the Kruskal–Wallis test. *Post hoc* comparisons of parametric variables using ANOVA were performed with Tukey’s test or the Games–Howell test, and *post hoc* comparisons of nonparametric variables according to the Kruskal–Wallis test were performed using the Steel–Dwass multiple comparison test. *P*-values < 0.05 indicated statistical significance.

## Results

### Number of rats used in this experiment

There were 24 female Sprague–Dawley rats used in this experiment. There were 6 rats in each group.

### Comparison of UBP and A-URE

**Fig. 1:**
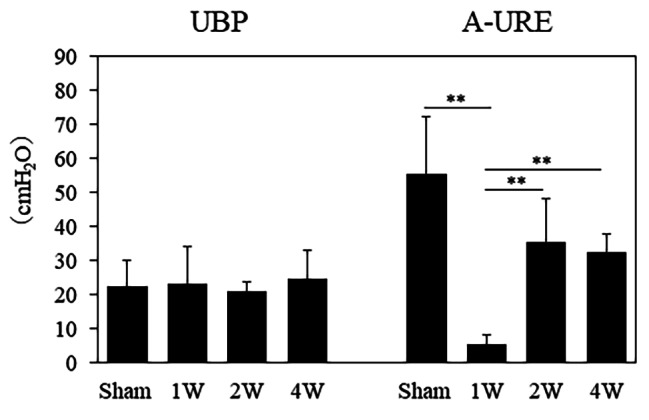
There were no significant differences in UBP among the sham, 1 W, 2 W, and 4 W groups. The A-URE values were significantly lower in the 1 W, 2 W, and 4 W groups versus the sham group (all *p* < 0.01). UBP, urethral baseline pressure; A-URE, amplitude of the urethral response to electrical stimulation; sham group, sham-operated group; 1 W group, 1 week after vaginal distention; 2 W group, 2 weeks after vaginal distention; 4 W group, 4 weeks after vaginal distention. Values are mean ± standard deviation. Statistical analysis ANOVA, Games-Howell test. **Indicates a statistically significant difference (*p* < 0.01)

There were no significant differences in UBP between the sham (22.3 ± 7.6 cmH_2_O), 1 W (23.2 ± 10.9 cmH_2_O), 2 W (20.9 ± 2.7 cmH_2_O), and 4 W (24.7 ± 8.1 cmH_2_O) groups. The A-URE was significantly lower in the 1 W group (5.2 ± 3.0 cmH_2_O) than in the sham (55.4 ± 17.0 cmH_2_O), 2 W (35.5 ± 12.6 cmH_2_O), and 4 W groups (32.5 ± 5.2 cmH_2_O) (all *p* < 0.01) (Fig. 1).

### Comparison of muscle wet weight /body weight

**Fig. 2:**
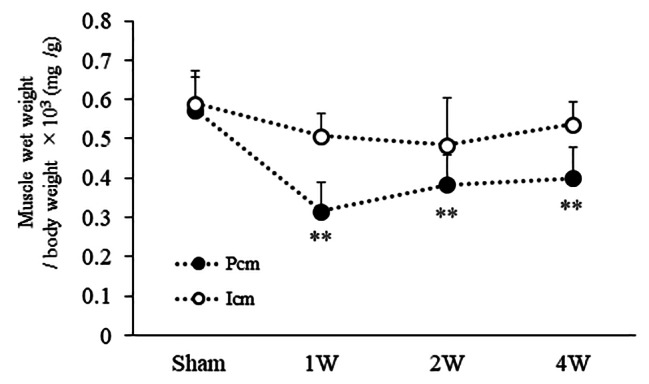
The muscle wet weight of the Pcm was significantly lower in the 1 W, 2 W, and 4 W groups versus the sham group (all *p* < 0.01). The muscle wet weight of the Icm did not differ significantly among the four groups. Pcm, pubococcygeus muscle; Icm, iliococcygeus muscle; sham group, sham-operated group; 1 W group, 1 week after vaginal distention; 2 W group, 2 weeks after vaginal distention; 4 W group, 4 weeks after vaginal distention. Values are mean ± standard deviation. Statistical analysis Tukey’s test. **Indicates a statistically significant difference (*p* < 0.01 versus the sham group)

Muscle wet weight / body weight of the Pcm was significantly lower in the 1 W (0.31 ± 0.07 mg/g), 2 W (0.38 ± 0.07 mg/g), and 4 W (0.39 ± 0.07 mg/g) groups than in the sham group (0.57 ± 0.08 mg/g) (all *p* < 0.01). The muscle wet weight of the Icm was not significantly different among the four groups (sham: 0.58 ± 0.08 mg/g; 1 W: 0.50 ± 0.05 mg/g; 2 W: 0.48 ± 0.11 mg/g; 4 W: 0.53 ± 0.05 mg/g) (Fig. 2).

### Comparison of fiber CSA

**Fig. 3:**
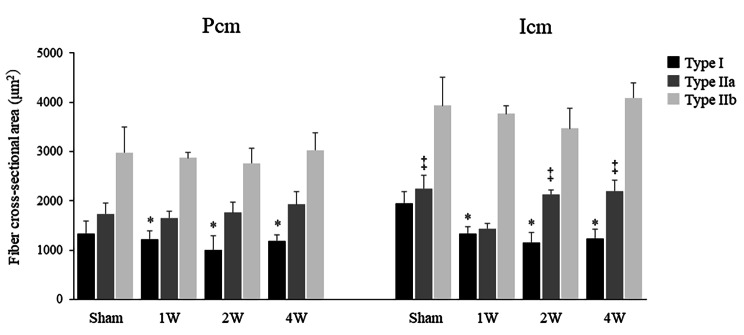
The CSA of Type I Pcm fibers was significantly lower in the 1 W, 2 W, and 4 W groups versus the sham group. The CSA of Type IIa and IIb fibers in the Pcm did not differ significantly between the four groups. The CSA of Type I fibers in the Icm fibers was significantly lower in the 1 W, 2 W, and 4 W groups versus the sham group. The CSA of Type IIa fibers in the Icm was significantly lower in the 1 W group versus the sham, 2 W, and 4 W groups (all *p* < 0.01). The CSA of Type IIb fibers in the Icm did not differ significantly among the four groups. CSA, cross-sectional area; Pcm, pubococcygeus muscle; Icm, iliococcygeus muscle; sham group, sham-operated group; 1 W group, 1 week after vaginal distention; 2 W group, 2 weeks after vaginal distention; 4 W group, 4 weeks after vaginal distention. Values are mean ± standard deviation. Statistical analysis ANOVA, Welch’s t-test and Steel–Dwass multiple comparison test. *Indicates a statistically significant difference (*p* < 0.05 versus the sham group); ‡Indicates a statistically significant difference (*p* < 0.01 versus the 1 W group)

The CSA of Type I fibers in the Pcm was significantly lower in the 1 W (1,223.1 ± 173.3 µm^2^, *p* = 0.02), 2 W (995.9 ± 295.6 µm^2^, *p* = 0.03), and 4 W (1,183.9 ± 126.7 µm^2^, *p* = 0.02) groups than in the sham group (1,337.2 ± 252.9 µm^2^). The CSA of Type IIa fibers (*p* = 0.16) and IIb fibers (*p* = 0.59) in the Pcm did not differ significantly between the four groups (Fig. 3).

The CSA of Type I fibers in the Icm was significantly lower in the 1 W (1,340.7 ± 137.0 µm^2^, *p* = 0.02), 2 W (1,158.4 ± 207.5 µm^2^, *p* = 0.03), and 4 W (1,238.9 ± 179.4 µm^2^, *p* = 0.02) groups than in the sham group (1,947.8 ± 235.5 µm^2^). The CSA of Type IIa fibers in the Icm was significantly lower in the 1 W group (1,438.5 ± 105.1 µm^2^) than in the sham (2,246.5 ± 265.1 µm^2^), 2 W (2,134.4 ± 85.2 µm^2^), and 4 W (2,201.2 ± 225.8 µm^2^) groups (all *p* < 0.01). The CSA of Type IIb fibers in the Icm was not significantly different among the four groups (*p* = 0.06) (Fig. 3).

### Changes in the ratio of types I, IIa, and IIb fibers in the Pcm and Icm

#### Pcm

**Fig. 4:**
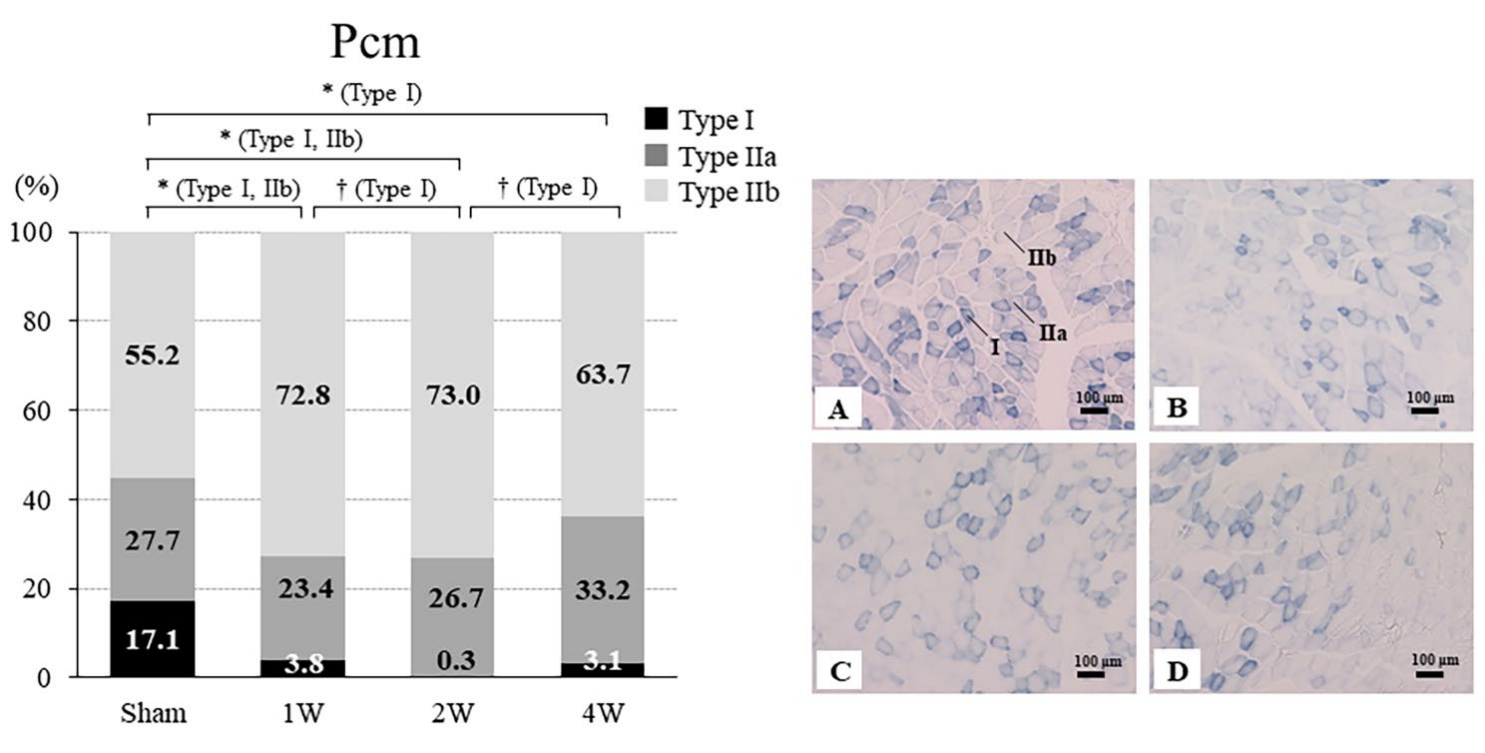
Ratios of Type I, IIa, and IIb fibers in the Pcm and representative SDH staining of the CSA of the Pcm from the sham, 1 W, 2 W, and 4 W groups. The ratio of Type I was significantly lower in the 1 W, 2 W, and 4 W groups versus the sham group. The ratio of Type I was significantly lower in the 2 W group versus the 1 and 4 W groups. The ratio of Type IIa did not differ significantly between the four groups. The ratio of Type IIb was significantly higher in the 1 and 2 W groups versus the sham group (*p* < 0.01). SDH-stained cross section of the rat Pcm: the sham group **(A)**, 1 W group **(B)**, 2 W group **(C)**, and 4 W group **(D)**. CSA, cross-sectional area; SDH, succinate dehydrogenase; Pcm, pubococcygeus muscle; sham group, sham-operated group; 1 W group, 1 week after vaginal distention; 2 W group, 2 weeks after vaginal distention; 4 W group, 4 weeks after vaginal distention. Values are mean ± standard deviation. Statistical analysis ANOVA, Steel–Dwass multiple comparison test, Tukey’s test. *Indicates a statistically significant difference (*p* < 0.05 versus the sham group); †Indicates a statistically significant difference (*p* < 0.05 versus the 2 W group). Scale bar = 100 μm

The ratio of Type I was significantly lower in the 1 W (3.8% ± 2.4%, *p* = 0.02), 2 W (0.3% ± 0.5%, *p* = 0.01), and 4 W (3.1% ± 2.7%, *p* = 0.02) groups than in the sham group (17.1% ± 3.4%). The ratio of Type I was significantly lower in the 2 W group versus the 1 W (*p* = 0.01) and 4 W (*p* = 0.04) groups. The ratio of Type IIa was not significantly different among the four groups (*p* = 0.20). The ratio of Type IIb was significantly higher in the 1 W (72.8% ± 11.4%) and 2 W (73.0% ± 9.9%) groups than in the sham group (55.2% ± 3.6%) (all *p* < 0.01) (Fig. 4).

#### Icm

**Fig. 5:**
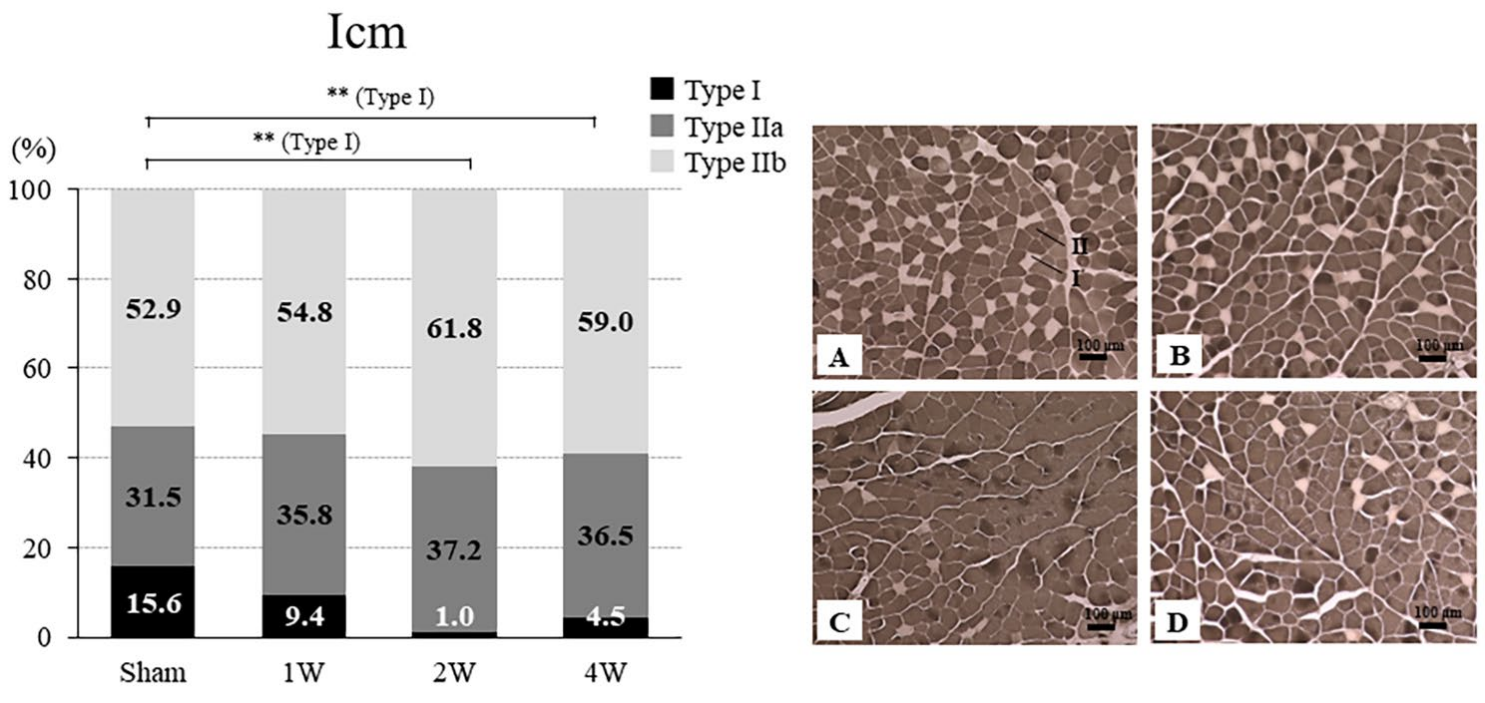
Ratio of Type I, IIa, and IIb fibers in the Icm and representative ATPase (pH 10.2) staining of the CSA of the Pcm from the sham, 1 W, 2 W, and 4 W groups. The ratio of Type I was significantly lower in the 2 and 4 W groups versus the sham group (*p* < 0.01). Ratios of Type IIa and IIb did not differ significantly between the four groups. ATPase (pH 10.2)-stained cross section of the rat Pcm: the sham group **(A)**, 1 W group **(B)**, 2 W group **(C)**, and 4 W group **(D)**. CSA, cross-sectional area; ATPase, myosin adenosine triphosphatase; Icm, iliococcygeus muscle; sham group, sham-operated group; 1 W group, 1 week after vaginal distention; 2 W group, 2 weeks after vaginal distention; 4 W group, 4 weeks after vaginal distention. Values are the mean. Statistical analysis ANOVA, Steel–Dwass multiple comparison test. **Indicates a statistically significant difference (*p* < 0.01 versus the sham group). Scale bar = 100 μm

The ratio of Type I was significantly lower in the 2 W (1.0% ± 1.1%) and 4 W (4.5% ± 3.1%) groups versus the sham group (*p* < 0.01). The ratios of Type IIa and IIb were not significantly different among the four groups (Fig. 5).

## Discussion

In this study, for the first time, evidence indicates that the composition of the pelvic floor muscles was altered in rat model of VD.

First, we evaluated the urethral function to confirm that VD simulates stress urinary incontinence after vaginal delivery. In this analysis, the intraurethral pressure (A-URE) reached its lowest value at 1 week after VD. Previous reports have confirmed that the A-URE reaches its lowest level after 4 days of VD, following the development of stress urinary incontinence [12]. A-URE was determined as the maximal pressure change from baseline in cmH_2_O which evaluates the dynamic function of urethral function. Clinically, reduction of A-URE In the rat model of VD may reflect the remaining damage in the external urethral sphincter dysfunction and urethral closure reflexes evident [3]. Therefore, our findings are consistent with those of a previous study showing that the VD can be used as a model of stress urinary incontinence after vaginal delivery.

Our date demonstrated that the muscle wet weight / body weight of the Pcm was significantly lower in the VD groups versus the sham group.

This observation suggests that we think that muscle atrophy occurred from the first week after VD because the Pcm is more severely damaged by VD, which is consistent with a previous study [15]. Regarding vaginal delivery, the Pcm is the most elongated among the pelvic floor muscles [4] and prone to injury after vaginal delivery [16]. The Pcm begins from the internal surface of the pubic bone immediately lateral to the vagina, while the Icm is located immediately lateral to the Pcm. The A-URE did not differ significantly between the 2 W, 4 W, and sham group, suggesting the recovery of urethral function in the 2 and 4 W groups. However, the Pcm showed persistent decrease even in these groups. These differences in the recovery state after VD suggest that urethral function may be restored more rapidly than the pelvic floor muscles.

In addition, our data further demonstrated that the CSA, Type I fibers in the Pcm and Icm were significantly lower in the VD groups. In a study examining the effects of denervation in male rats, it was found that removal of the somatomotor branch from the pelvic nerve innervating the Pcm reduced the CSA of Pcm, [17] and our study also found similar results. In rats, the pelvic nerve is bifurcated; it includes a viscerocutaneous (sensory) branch, receiving information from the pelvic viscera and the midline perineal region, as well as a somatomotor (muscular) branch, which innervates the Pcm and Icm [18]. In humans, the Pcm and Icm are innervated by the pudendal nerves for both sensation and movement. Stretching of the pudendal nerve during vaginal birth in humans exhibits up to 35% strain [19]. VD in rats dilates the distal part of the vagina and the somatic nerves (e.g., dorsal nerve of the clitoris and sacral plexus) that run along it [20]. 

Another study examining the CSA of the soleus and plantaris muscles in rats after sciatic nerve injury found that the CSA of Type I fibers in the soleus muscle and the CSA of Type II fibers in the plantaris muscle atrophied early after injury. This reflects the fact that the denervation process exerts different effects on the two types of fiber in slow- and fast-twitch muscles in the early stages [21]. The relatively large reductions in the CSA of Type I fibers in the Pcm observed in the present study are consistent with an atrophic effect that is more pronounced in the predominant type of fiber within the muscle [22]. 

Regarding the muscle composition of Pcm and Icm, the VD group showed a shift in muscle fiber phenotype from slow to fast muscle. Numerous studies have demonstrated a decrease in slow-twitch muscle fibers and an increase in fast-twitch muscle fibers after denervation [23]. The present evidence is consistent with these results. Denervation reduces the ability of muscles to generate force [24]. Electromyography has shown that pelvic floor muscles activation patterns are altered in women with stress urinary incontinence versus healthy controls, with delayed activation, shorter activation periods, a lack of response, and paradoxical inhibition [25]. These findings suggest that vaginal delivery may alter the composition of the pelvic floor muscles, leading to a decrease in slow-twitch muscle response and overall pelvic floor muscles contractility.

Pathological conditions of stress urinary incontinence include urethral hypermobility and intrinsic sphincter deficiency. Urethral hypermobility causes opening of the urethra during abdominal pressure loading as a result of the anatomical conditions of the female pelvic floor, childbirth, and age-related pelvic floor disorders, which cause the urethra to droop vaginally. Intrinsic sphincter deficiency is a disorder of the urethral sphincter that causes urinary incontinence, as the bladder neck and proximal urethra remain open even at rest. In the current VD model, stress urinary incontinence was attributed to urethral hypermobility. The levator ani muscle increases intraurethral pressure and is involved in urinary continence, which contributes to urinary continence through the instantaneous contraction of fast-twitch muscles during increased abdominal pressure. According to our data, VD alters the composition of the Pcm and Icm and results in atrophy of these muscles. These effects lead to reduced contractility of slow-twitch muscles, which in turn leads to stress urinary incontinence symptoms.

This study has several limitations. Firstly, the composition of the pelvic floor muscles differs between humans and rats. The predominant muscles in rats and humans are slow- and fast-twitch muscles, respectively [26]. Secondly, the long-term effects with respect to the composition of the pelvic floor muscles after VD were not examined.

Future research should elucidate the mechanism of stress urinary incontinence that persists for more than 3 months postpartum with respect to histological examination of the pelvic floor muscles.

## Conclusions

Following VD in rats, muscle atrophy and changes in muscle composition in the pelvic floor muscles were observed even after improvements in urethral function. These results may enhance our understanding of the pathogenesis of stress urinary incontinence after VD.

## Data Availability

The datasets used and/or analyzed during the current study are available from the corresponding author on reasonable request.

## Abbreviations

A-URE: Amplitude of the urethral response to electrical stimulation
CSA: Cross-sectional area
Icm: Iliococcygeus muscle
Pcm: Pubococcygeus muscle
UBP: Urethral baseline pressure
